# Skeletal Muscle Adiposity Is Associated With Lower Cognitive Function In Older Cancer Survivors

**DOI:** 10.1093/geroni/igaf122.3208

**Published:** 2025-12-31

**Authors:** Brendan McNeish, Iva Miljkovic, Anne Newman, Caterina Rosano

**Affiliations:** University of Michigan, Ann Arbor, Michigan, United States; University of Pittsburgh, Pittsburgh, Pennsylvania, United States; University of Pittsburgh, Pittsburgh, Pennsylvania, United States; University of Pittsburgh, Pittsburgh, Pennsylvania, United States

## Abstract

Cancer is associated with accelerated aging and changes in both muscle and cognition. In cancer-free older adults, an emerging association between muscle adiposity and cognition has been observed; however, this relationship has not been investigated in older cancer survivors. This study aimed to evaluate the association between skeletal muscle adiposity and cognitive function in older cancer survivors. This study included 75 cancer survivors who developed cancer within the first five years of the study (age range: 69–79; 65% men; 31% Black; 41% prostate cancer; 21% breast cancer). Thigh intermuscular fat area, a measure of skeletal muscle adiposity, was obtained via computed tomography at Year 6. Cognitive function was assessed using the Digit Symbol Substitution Test (DSST) and the Mini-Mental Status Exam (MMS) at Year 10. Multivariable models adjusted for demographic factors (age, gender, race, education) and thigh muscle area at Year 6. Sensitivity analyses separately adjusted for dementia risk factors, including diabetes, hypertension, APOE4 allele, physical activity, and adiposity measures at Year 6 (BMI, abdominal visceral and subcutaneous adiposity, and total body fat mass). Greater thigh intermuscular fat area was associated with lower DSST scores (B= -0.298, p < 0.05) and lower MMS scores (B= -0.195, p < 0.05) at Year 10. These associations remained significant and were not attenuated by dementia risk factors or other adiposity measures. Higher skeletal muscle adiposity is independently associated with lower cognitive function in older cancer survivors. Further research is needed to confirm this relationship and explore the underlying biological mechanisms.

